# Teeth, Sex, and Testosterone: Aging in the World's Smallest Primate

**DOI:** 10.1371/journal.pone.0109528

**Published:** 2014-10-29

**Authors:** Sarah Zohdy, Brian D. Gerber, Stacey Tecot, Marina B. Blanco, Julia M. Winchester, Patricia C. Wright, Jukka Jernvall

**Affiliations:** 1 Institute of Biotechnology, University of Helsinki, Helsinki, Finland; 2 Centre ValBio Research Station, Ranomafana, Ifanadiana, Madagascar; 3 Department of Environmental Studies, Emory University School of Public Health, Emory University, Atlanta, Georgia, United States of America; 4 Colorado Cooperative Fish and Wildlife Research Unit, Department of Fish, Wildlife and Conservation Biology, Colorado State University, Fort Collins, Colorado, United States of America; 5 School of Anthropology, University of Arizona, Tuscon, Arizona, United States of America; 6 Duke Lemur Center, Durham, North Carolina, United States of America; 7 Department of Anthropology, Stony Brook University, Stony Brook, New York, United States of America; Northern Illinois University, United States of America

## Abstract

Mouse lemurs (*Microcebus* spp.) are an exciting new primate model for understanding human aging and disease. In captivity, *Microcebus murinus* develops human-like ailments of old age after five years (e.g., neurodegeneration analogous to Alzheimer's disease) but can live beyond 12 years. It is believed that wild *Microcebus* follow a similar pattern of senescence observed in captive animals, but that predation limits their lifespan to four years, thus preventing observance of these diseases in the wild. Testing whether this assumption is true is informative about both *Microcebus* natural history and environmental influences on senescence, leading to interpretation of findings for models of human aging. Additionally, the study of *Microcebus* longevity provides an opportunity to better understand mechanisms of sex-biased longevity. Longevity is often shorter in males of species with high male-male competition, such as *Microcebus*, but mouse lemurs are sexually monomorphic, suggesting similar lifespans. We collected individual-based observations of wild brown mouse lemurs (*Microcebus rufus*) from 2003–2010 to investigate sex-differences in survival and longevity. Fecal testosterone was measured as a potential mechanism of sex-based differences in survival. We used a combination of high-resolution tooth wear techniques, mark-recapture, and hormone enzyme immunoassays. We found no dental or physical signs of senescence in *M. rufus* as old as eight years (N = 189, ages 1–8, mean = 2.59±1.63 SE), three years older than captive, senescent congeners (*M. murinus*). Unlike other polygynandrous vertebrates, we found no sex difference in age-dependent survival, nor sex or age differences in testosterone levels. While elevated male testosterone levels have been implicated in shorter lifespans in several species, this is one of the first studies to show equivalent testosterone levels accompanying equivalent lifespans. Future research on captive aged individuals can determine if senescence is partially a condition of their captive environment, and studies controlling for various environmental factors will further our understanding of senescence.

## Introduction

Wild animals have to contend with high levels of extrinsic sources of mortality, such as predation and starvation, in contrast with captive animals and the majority of humans living in industrial societies. Consequently, a common generalization is that senescence as observed in captive animals should rarely or never be observed in wild populations [1], [2], [3]. As Rose (1991) puts the argument, “it is doubtful that many individuals [in wild populations] would remain for study at the age at which laboratory populations exhibit ageing” (pg. 21). While it is likely that the number of senescent individuals in wild populations is reduced by high pre-senescent mortality rates, a growing body of research shows senescence-related changes in life-history traits in wild animal populations [4], [5], [6]. It is thought that captive conditions, which eliminate extrinsic mortality factors, such as predation, disease, starvation, and environmental stressors, allow species to live longer in captivity [7]. At the same time, lower requirements for strenuous physical activity [8] and mental function required for foraging and avoiding predation in captivity [9], [10] may impact longevity and senescence in various ways. Thus, studies of wild and free ranging animals are helpful for understanding the underlying mechanisms affecting longevity and senescence [11], and complement results gained from captive studies. By comparing the longevity of captive and wild animals, we can begin to understand how the physiological and behavioral demands of captive and wild environments affect the aging process.

Captive research has demonstrated that mouse lemurs (*Microcebus* spp.) are an excellent model for understanding the behavioral and physiological correlates of human aging [12], [13], [14]. Although they weigh a mere 30–80 grams, mouse lemurs have been found to live up to 12.3 years in captivity, or more than six times longer than mammals of similar body size, such as mice or shrews [15]. Captive mouse lemurs over the age of five years have been shown to develop many symptoms of senescence, including neurodegeneration [14], slowing of motor skills and activity levels [16], changes within the biological clock [17], reduced cognitive and memory function [18], chemosensory responsiveness decline [19], and graying of hair and cataracts [12], [13], [20]. These patterns have led to postulations that extrinsic mortality factors experienced in the wild would limit the longevity of wild animals, such that individuals would rarely live past four years of age [21], [22], [23]. Thus, physiological changes and diseases related to senescence would not be observed in wild mouse lemurs. The longevity of wild mouse lemurs and the prevalence of physical manifestations of senescence in individuals are unknown.

Longitudinal studies of the effects of aging in wild animals are uncommon with only a few studies being conducted on small mammals. The technical challenges of identifying and following small animals long enough to obtain an accurate age structure has likely limited the number of these studies [11], [24]. Whereas mark-recaptures of individuals provide minimum estimates of ages, additional methods are required to estimate the ages of captured individuals to obtain accurate age tabulations for a population. Tooth wear analysis has been used previously in combination with mark-recapture studies to obtain age structure, but due to the short life-history of most small mammals, mark-recapture and dental wear studies have not occurred simultaneously; this work has been limited to studies of relatively large animals in which methods have been developed [25], [26], [27], [28].

Determining the longevity of a species is also complicated by the often-observed difference in the rate of aging between males and females [29]. Within polygynous or polygynandrous vertebrates, including humans, the average lifespan of females often exceeds that of males [30], [31]. A number of proximate explanations for this difference include 1) the costs of growing large, where sexual-size dimorphism leads to lower survival of males [32], 2) male propensity for riskier behaviors through dispersal and territorial defense [33], [34], and 3) lower immunocompetence due to higher levels of androgens (e.g., testosterone) often found in males, which may lead to disease and infection [35], [36]. Higher production and secretion of testosterone levels in males from puberty until death, as well as seasonally and during competitive interactions, have been suggested to result in shorter lifespans for males, compared to females [37]. In contrast, sexually monomorphic species may be expected to have no or only a small difference in sex-biased survival compared to sexually dimorphic species [31], when males and females also behave similarly (i.e., aggression rates and dispersal are not sex-biased). Based on evidence from other polygynandrous species, high male-male competition and male-biased dispersal would suggest that mouse lemur longevity would differ by sex [30], [38].

The goal of this study was to develop a reliable method of aging small mammals, so as to better understand aging and senescence in one species of wild mouse lemurs (i.e., *Microcebus rufus*). We used a combination of mark-recapture, high-resolution tooth wear analysis and fecal sampling to investigate mouse lemur 1) lifespan, and 2) signs of senescence. To further understand these patterns, we also investigated sex differences in 3) age-dependent survival and 4) testosterone. We predicted that wild brown mouse lemur longevity would be limited to 4 years of age and that no signs of senescence would be observed. Based on studies of wild mammals, predictions regarding sex differences in longevity are difficult because mouse lemurs have high levels of male-male competition for mates and mate polygynandrously, but are monomorphic. Lastly, we predicted that if males did not have shorter lifespans, they would also not have higher testosterone levels than females.

## Materials and Methods

### Ethics Statement

All research protocols were reviewed and approved by the government of Madagascar. Sample collection in Ranomafana National Park was approved by Madagascar National Parks under permit numbers #115/10 MEF/SG/DGF/DCB.SAP/SCBSE 96 and #215/08 MEFT/SG/DGEF/DSAP/SSE. Research protocols were also reviewed and approved by the University of Helsinki's institutional animal use rules and regulations board, and the Stony Brook University Internal Review Board and Institutional Animal Care and Use Committee (IACUC ID#2009-1608 and IACUC #2007-1597). No anesthetics were necessary (as deemed by two licensed veterinarians) for this research after 2008, as methods were minimally invasive, and animals were captured only briefly and returned to the wild immediately after data collection. All research protocols adhere to the American Society of Primatologists Principles for the Ethical Treatment of Non Human Primates.

### Study species, site, and trapping

We established a long-term trapping system from 2003–2010 in central-southeast Madagascar to study brown mouse lemurs (*Microcebus rufus*) in Ranomafana National Park (RNP; 47° 18′–47° 37′E and 21° 02′–21° 25′S). RNP includes 43,500 hectares of mostly montane rainforest [39]. Madagascar's eastern rainforest environment is highly variable, with low seasonality and high inter-annual variation in precipitation [40], and thus, variable and unpredictable seasonal fruiting. Brown mouse lemurs are an arboreal, nocturnal, seasonally breeding, female-dominant, monomorphic, polygynandrous, strepsirrhine primate. Their diet consists of a mixture of fruit, seeds, flowers, and insects [41]. To deal with their hyper-variable environment, mouse lemurs are known to use torpor in response to low food availability [42], though whether males and females equally use torpor to reduce energy expenses is unresolved [42], [43], [44].

Starting in 2007, we used a systematic trapping grid, where fifty Sherman traps (XLR, Sherman Traps Inc., FL) were set in pairs at ≈50 m intervals along two transects that covered an area of 7 km^2^. This area is locally known as Talatakely, which ranges in elevation from 835–1116 m, and was selectively logged from 1986–1989 [45]. Traps were set at 1–3 m off the ground and baited nightly with fresh fruit and were set consecutively from August-December. Our sampling period spanned the end of the cold season to the beginning of the rainy season, including the mouse lemur breeding season. Captured mouse lemurs were individually scanned (using an AVID Powertracker VI) for a microchip (FECAVA Eurochips,Vetcare, FI), sexed, weighed, and measured under infrered lighting, and then released back into the forest at their capture location. Individuals without a microchip were given one (FECAVA Eurochips,Vetcare, FI). Non-primate captures were released on site. Fresh fecal samples were collected from 56 males (339 samples) and 40 females (201 samples) for hormone analysis. Mouse lemurs were examined for external signs of senescence, including the graying of hair and ocular pathologies such as cataracts. We examined individuals' eyes by capturing images using an infrared Sony HDX-10 video camera (Figure S1 in File S1).

### Dental impressions and tooth wear analysis

While ages of juveniles (<1 year) were known with certainty and tracked throughout our study, older individuals' ages were unknown and required estimation. We used dental impressions to estimate ages of captured individuals. We began by propping the mouths of captured animals open so that the lower tooth rows could be cleaned using human infant interdental brush tips and sterile water. Teeth were dried by pumping air through a disposable 1 ml pipette, and impression material (Express, fast set, 3M Dental Products) was applied and left to set for two minutes.

Dental molds were set on glass slides, lightly brushed with talcum powder and sprayed with graphite to improve visualization. To obtain consistent measurements between molds, dental molds were positioned on glass slides to maximize the views of both buccal and lingual sides of the crown, thereby providing an occlusal view of the whole crown. Using an AX70 Olympus microscope (Melville, NY) camera, occlusal view photos were taken of the entire tooth row at 9.4 magnification, and the molar row at 15 magnification. We made linear measurements of exposed dentine on the metaconid and the hypoconid cusps of the second mandibular molar. The metaconid was measured anterio-distally and the hypoconid bucco-lingually [File S1]. Whereas other cusps also showed wear, the metaconid and hypoconid were the most consistently worn in these small teeth (Figure S2 in File S1). Dental wear was calculated as the average of these two measurements relative to the length of the second mandibular molar. In addition, we scanned dental impressions using a Nextec Hawk 3D laser scanner at 20 µm resolution to better visualize wear. From these scans, we created digital elevation models (DEMs) and calculated the Orientation Patch Count (OPC), which is a measure of dental complexity and senescence of dental function. OPC values were calculated following previous studies [46], [47]; comparable values on whole tooth rows were calculated by down-sampling DEMs into a grid having 50 data rows along the tooth length.

**Figure 1:**
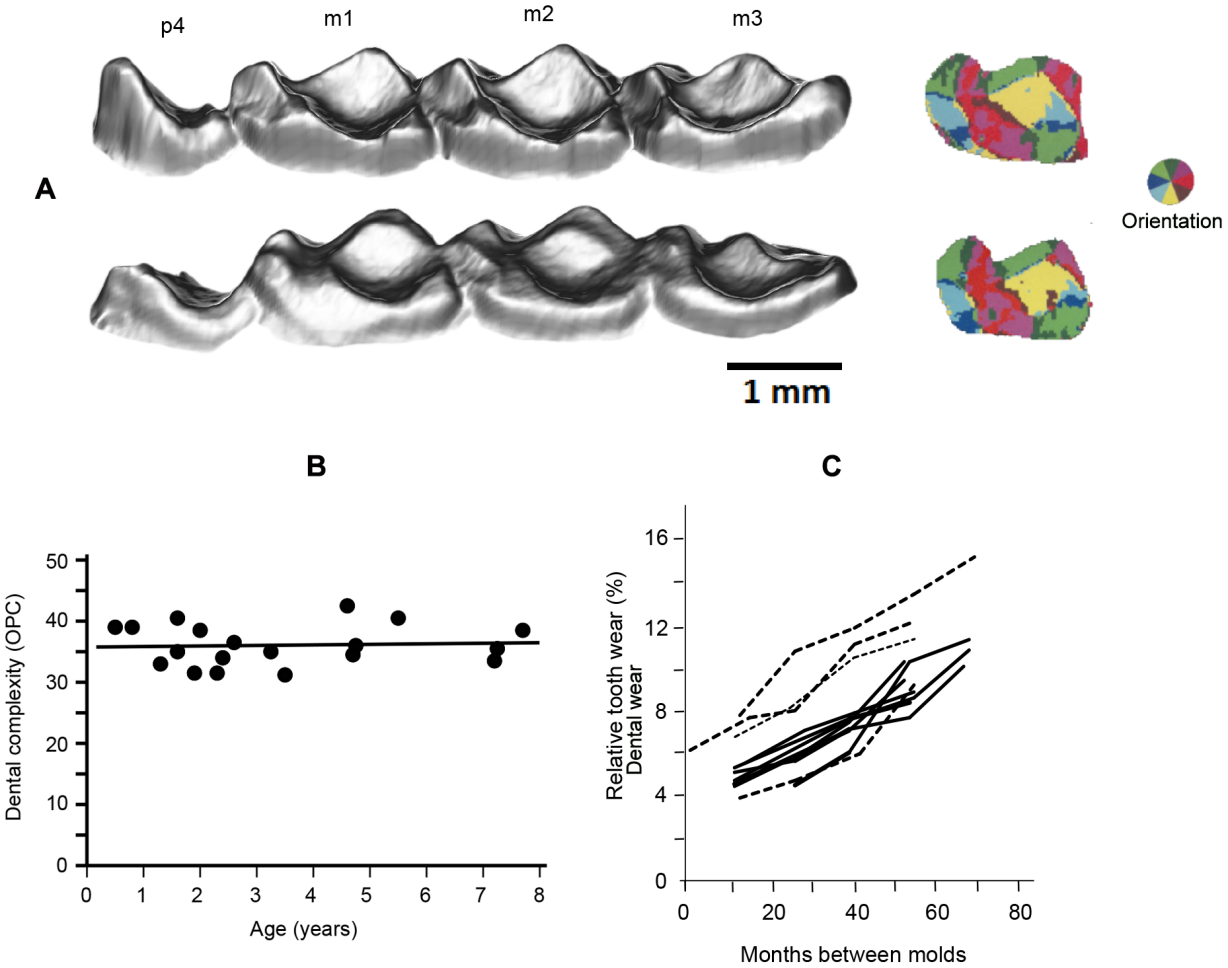
Aging of mouse lemurs using high-resolution tooth wear analysis. A: Obliquely lingual view of tooth rows and dental orientation patch count (OPC) of a one year-old (top) and seven year-old (bottom); p = pre-molar, m = molar. The darker shading indicates wear. B: OPC by age. C: Tooth wear rates of individual males (solid lines) and females (dashed lines) captured four or more consecutive years.

To estimate mouse lemur age, we constructed a regression model; the slope was calculated as the mean wear rate of all individuals with at least three consecutive captures. Because brown mouse lemurs are highly seasonal breeders, with the majority of individuals born in December [48], the regression intercept was calculated by taking the mean wear from first-time captured individuals showing the least wear; these individuals were estimated to be 9.5 months of age. To estimate senescence in dental function, we used the OPC measure of dental complexity [46], calculated from 3D scale of the second lower molars (Figure 1a). We performed all data analyses using SAS statistical software, version 9.2 (SAS Institute Inc., Cary, NC, USA).

### Hormone assay procedures

Collected fecal samples were heated and desiccated at 70°C for 2–3 days and kept in silica gel at room temperature until extraction. Fecal testosterone (T) analyses were performed as previously described [49], [50]. Desiccated fecal samples were ground into a fine powder using a mortar and pestle and weighed out to 0.1 g. Fecal steroids were isolated using ethanol water extractions, with 2.5 ml of 100% ethanol and 2.5 ml of distilled H20. To release conjugated steroids, 1 ml of the 5 ml extract was added to 4 ml of ethyl acetate, then homogenized and centrifuged. The ethyl acetate was then aspirated off and suspended in 1 ml of 30% methanol. T was separated from the fecal extract by celite chromatography using the System I technique [49], [51]. 100 µl of the separated sample was aliquoted onto 96-well microtitre plates and put through enzyme immunoassay (EIA) using T∶HRP as a secondary antibody (R156, Munro, University of California, Davis). Absorbance was read at 415 nm (background absorbance of 570 nm subtracted) on a Spectramax 340 (Molecular Devices Corporation, Sunnyvale, California, U.S.A.). Parallelism was determined through serial dilutions of the fecal pool along the standard curve. In addition to running a standard curve, fecal pool samples were run in duplicate on each assay in order to establish a mean intra- and interassay coefficient of variation (CV) for T. Mean steroid recovery was 75%. Accuracy assessment was within acceptable parameters (above 90%). A mean percent accuracy was determined for T (98.91±3.06%) assays. Pooled samples were parallel to the standard curve for T (t = 1.48; df = 24). In addition, mean intra- and interassay CV values were found to be within acceptable limits for both low (no difference in slope; T: intra = 6.9%, inter = 34.2%) and high (no difference in slope; T: intra = 4.7%, inter = 14.9%) pools.

We evaluated whether there were differences in T levels by age and sex using a generalized linear mixed effect model, where the response variable was Gamma distributed. We considered *age* and *sex* as fixed effects and treated *individual* as a random effect, because repeated measurements were made of individuals.

### Survival analysis

We estimated brown mouse lemur annual survival probability using a closed robust capture-recapture model with the Huggins estimator implemented in program MARK [52], [53], [54]. We considered four primary years of sampling (2007–2010) and limited data to October. During this time, most of the population is active and likely not in torpor, nor moving a lot as during the breeding season, such that the population could be considered closed to demographic and geographic changes. We tested population closure within each primary period using the Otis closure test [55]. We considered an a priori set of 24 models that represented biologically-driven hypotheses. Survival was hypothesized to be constant, vary by sex, age, and year, linear and quadratic age trend, or mixed combinations. Age was determined by the above mentioned dental measurements. Detection probability was hypothesized to vary by year, age, sex, individual heterogeneity, sampling occasion, or mixed combinations. Heterogeneity was modeled using a finite mixture model [56]. We evaluated model parsimony using Akaike's Information Criterion with a small sample bias correction (AICc) [57]; parameter estimates were model-averaged to incorporate model selection uncertainty. We used a series of goodness-of-fit tests from the program RELEASE to evaluate whether our most general model was appropriate for our data ([58]; File S1).

## Results

A total of 420 dental impressions were taken from the lower right mandibular tooth rows of 189 unique individuals. 270 age estimates were calculated (Table S1, Table S2 in File S1). The numbers of individuals by year (2003–2010) for which we obtained age estimates were 3, 12, 21, 15, 36, 54, 69, and 60, consecutively. For 23 individuals captured three or more consecutive years during the total duration of the study, the regression slopes of wear rates were calculated (r^2^ = 0.546–0.997; Table S3 in File S1), and the mean slope was used to calculate ages for all individuals. We found that the estimated ages for 92.7% of individuals were the same (48.6%) or greater (51.4%) than the minimum possible ages based on recapture data alone (Table S4 in File S1). Of the 17 individuals that had predicted ages younger than minimum possible age according to recapture data, the estimated ages were too young by only one year or less.

We found that the oldest mouse lemurs were estimated to be eight years of age. No individuals showed any external signs of aging (graying of hair or cataracts). We observed a single mouse lemur with ocular pathology (Figure S1 in File S1), in which the eye atrophied in the following year. Despite observing tooth wear, we found that OPC values did not change with age (N = 19, *P* = 0.81; Figure 1b). Because the coronal enamel between cusps is still retained in the oldest individuals, further wear is needed to reach dental senescence; thus, dental function is likely to be retained into an advanced age in wild mouse lemurs. Dental wear in individuals captured four or more consecutive years shows relative consistency with no difference between the sexes (T = 0.66, *P* = 0.52; Table S5 in File S1, Figure 1c.).

**Figure 2:**
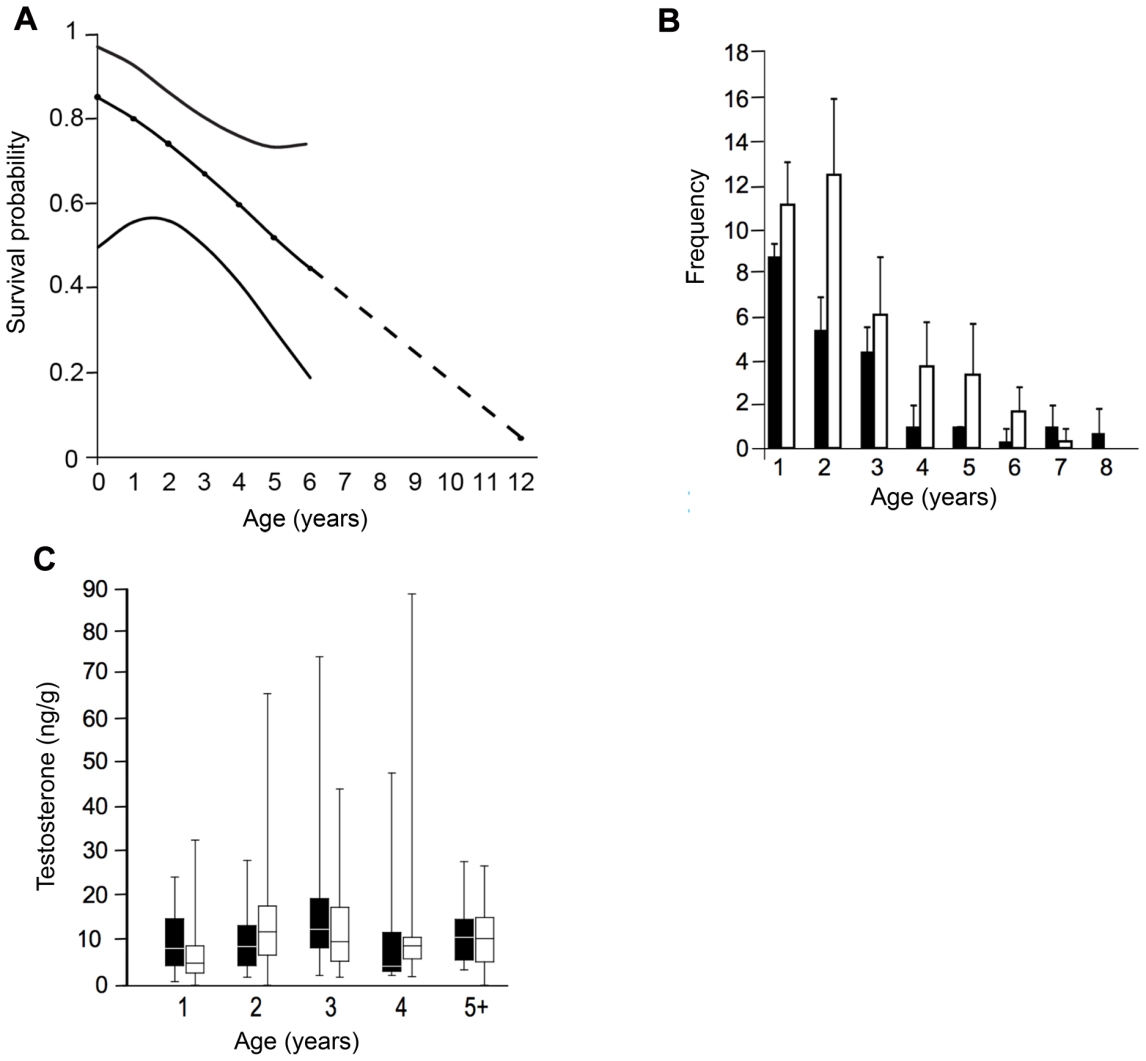
Estimates of survival, frequency of captures, and testosterone levels varying by age. A: Age dependent survival probability (dashed line) and 95% profile-likelihood confidence intervals. B: The frequency of female (black) and male (white) mouse lemurs included in the study with standard error bars by age. C: Fecal testosterone values for male (black) and female (white) mouse lemurs. Boxes enclose 50% of observations; the median is indicated with a horizontal bar, whiskers denote range.

We found our data to fit the most general survival model [File S1] and detected no violation of the closure assumption (Table 1). With both sexes combined, our most parsimonious model indicated that survival varied linearly by age (on the logit-scale; Figure 2a), whereas recapture probability was heterogeneous and varied between the sexes; however there was considerable model selection uncertainty (Table S6 in File S1). We found no differences in age-dependent survival between the sexes (β_sex_ = −0.44±0.64 SE, Table S7 in File S1). Both sexes lived beyond 4 years with 16% of individuals surviving past the predicted maximum age of wild mouse lemurs (Figure 2b).

**Table 1:** Summary of capture/recaptures and tests of population closure for *Microcebus rufus* capture history in October 2007–2010.

Primary Period	No. Secondary Periods	Unique Individuals	Total Captures	Recaptures	Closure Test *Z*	Closure Test *P*
2007	32	46	117	71	2	0.977
2008	33	40	120	80	−1.019	0.154
2009	31	32	87	55	0.848	0.801
2010	33	31	97	66	−0.690	0.245

The mean testosterone (T) values were 12.87±9.40 ng/g for females and 12.11±9.79 ng/g for males, with no significant difference in T values between the sexes (F(1,81) = 0.02, *P* = 0.90, Table S8 in File S1). Furthermore, in both sexes T levels did not decrease with age (*P* = 0.93, Figure 2c).

## Discussion

Our tooth-wear methods accurately estimated mouse lemur age. The estimated ages of known-aged individuals were mostly correct or underestimated by 1 year, such that there is a small underestimation bias. This level of accuracy was likely achievable because our study spanned many years and covered a large population, allowing us to recapture many individuals multiple times. When animals cannot be recaptured over a long period of time this tooth-wear method may need to be adjusted to accommodate shorter intervals, otherwise a greater estimation bias in age may occur. We believe our method will be helpful in expanding the study of senescence and aging beyond large-bodied animals. Investigating small mammal aging is important to understanding the influence of body size on the processes of longevity and senescence, as well as whether environmental effects are consistent across allometric scales.

We found that wild brown mouse lemurs (*M. rufus*) lived to at least eight years of age, which approaches captive longevity records of 12.3 years for *M. murinus* [15]. Based on the wear rates of the between-cusp coronal enamel, we suspect some individuals' teeth could remain functional beyond 10 years in the wild. This suggests that dental senescence is not what precludes individuals from living beyond 10 years. Previous suggestions that wild mouse lemurs would not live beyond four years of age [13], [22] are thus likely to be underestimates. In our population, we found that 16% of the individuals lived beyond 4 years of age. We note that at least two factors may have artificially increased the proportion of older individuals in our data, relative to the true population age structure. First, older individuals may be more experienced about their home range than younger individuals, and thus occupy traps more readily. However, our analysis of recaptures within each trapping season showed no increase in frequency of captures with age (Figure S3 in File S1), nor was detection probability by age supported by model selection in the survival analysis. Second, because our first time captures were caught at approximately 9.5 months after birth, our data lack individuals that died during their first winter. In order to consider these individuals in the age structure, we tabulated the maximum possible number of individuals in the first year age cohort by assuming that each female captured the previous year had successfully raised two infants, which is the average litter size in brown mouse lemurs. Of these individuals, we assumed 1 minus our mean survival probability of one- to two-year-olds were not alive to be sampled. After incorporating these individuals, the proportion of the population ≥4 would decrease to 12%. Overall, we found that 12–16% of our wild *M. rufus* population lived longer than captive data for *M. murinus* suggested.

We found no physical signs of senescence (graying of hair, cataracts, or dental) in any wild individual. However, as this study was conducted to maximize the potential of single time capture events to study aging in the wild, we were unable to document many of the gradual physiological symptoms of senescence documented in captive mouse lemurs (*M. murinus*), such as neurological state and brain condition [14], motor skills and activity levels [16], changes within the biological clock [17], reduced cognitive and memory function [18], and declines in chemosensory responsiveness [19]. Similarly, there is no captive evidence of dental senescence or lack thereof. Therefore, our results do not provide information about physiological senescence in wild (*M. rufus*) that can be directly compared to senescence in captive mouse lemurs (*M. murinus*), but do suggest that wild *M. rufus* have the potential to live well beyond the age at which physiological symptoms of senescence begin to occur in captive *M. murinus*. Here, we reveal that wild mouse lemur (*M. rufus*) longevity approaches that of captive *M. murinus* records; however, further research using identical measures of senescence will help to reveal whether patterns of physiological senescence occur consistently across the genus and in both captive and wild conditions. Primates have slow life histories and long lifespans relative to mammals of comparable body masses, perhaps due to their increased brain size [3]. As small primates that have these traits, there are many potential reasons that may explain why wild mouse lemurs are long-lived in the wild. One reason may be that mouse lemurs are capable of predator avoidance strategies that non-primate arboreal mammals may not have, due to their relatively large brain size, a trait typical among primates. Nocturnality and arboreality have been proposed as mechanisms of predator avoidance, and mouse lemurs are both nocturnal and arboreal, allowing them to avoid diurnal and terrestrial predators. Another potential mechanism behind long lifespan in wild mouse lemurs may be their ability to enter hibernation/torpor, a trait unique among primates. During hibernation metabolic rate is lowered which conserves energy and slows down the rate of cellular reproduction. Recent work has shown that this sort of thermoregulation has the ability to increase survival in mammals, and has been linked with slow life histories [59], [60]. Hibernation/torpor may also be differentially influenced by breeding patterns, seasonality, and resource acquisition, and therefore has the potential to play a role in varying patterns of longevity in different mouse lemur species. While hibernation may provide a potential explanation for long lifespan in wild mouse lemurs, we would have expected dental wear rates to reveal patterns of sex-specific hibernation if it occurs in this population. Since females are thought to hibernate for 6 months of the year, we would have expected to see females experiencing half the annual wear of males. We did not find any sex differences in dental wear; however, we did find a few individuals with predicted ages less than the minimum possible ages (based on longitudinal trapping data), which may reflect hibernation in those individuals. Nonetheless, there was no sex-biased pattern of tooth wear, and only very few individuals had these slow wear rates. This result suggests that perhaps there is no sex bias in hibernation in brown mouse lemurs, but that only individuals that can attain a large enough body mass can undergo the process of torpor/hibernation.

This study was conducted in just one of many wild species of mouse lemur in a rainforest environment. Wild mouse lemur species in dry and spiny forest habitats may not have the same ability to live long in the wild. Other species of wild mouse lemurs experience differences in patterns of breeding, seasonal torpor, and seasonality, factors that may affect their life histories [44]. For example, *Microcebus murinus*, the species extensively studied in captivity, exhibits sex differences in the duration of torpor and the extent to which torpor is initiated in the wild [44]. Furthermore, differences in seasonality, predator exposure, and other environmental factors may play a role in the reduction or extension of longevity. Future research on additional *Microcebus* species in the wild may help to elucidate seasonally-linked longevity and survival patterns in mouse lemurs.

Another potential explanation for the presence of old mouse lemurs in the wild may be natural selection. There is the possibility that the “aged” individuals we documented in the wild may just represent a subset of aged individuals that survive in captivity without experiencing any symptoms of senescence. However, there are no published statistics on the percentage of elderly mouse lemurs in captivity that are free from age-related pathologies, so whether or not this explains the number of “aged” individuals in the wild is yet to be seen, but further information on the matter may provide an explanation for the number of aged individuals found in the wild.

The survival rates and presence of aged males in our study indicate that mouse lemurs do not conform to the typical polygynandrous pattern of lower male survival. Polygynandrous male vertebrates are typically involved in intense male-male competition and experience reduced survival and lifespan and earlier senescence than females [30], a pattern found also in polygynandrous non-human primates [61]. The lack of difference between male and female testosterone levels and consistent testosterone levels among differently-aged mouse lemurs is in contrast with the decreasing testosterone levels in aging humans and captive mouse lemurs [15], [62]. We suggest that the combination of sexual monomorphism and female dominance characterizing most lemurs [63], [64] is manifested in the mouse lemur as the lack of difference between testosterone levels between sexes, and ultimately as comparable survival of males and females. This is one of the first studies to demonstrate that equivalent testosterone levels between sexes accompany equivalent lifespans, though more work is necessary to determine if both sexes have low testosterone levels or elevated testosterone levels, if sex-differences become apparent during different seasons, and if testosterone and lifespan are causally related.

## Conclusions

Natural processes that affect animal behavior can influence patterns of longevity and senescence in mouse lemurs and maybe other primates (e.g., humans). Our results provide evidence that senescence in captive animals that are used to study human aging and disease does not reflect complex natural processes, and suggest that elements of physical activity and mental function required for complex behaviors (e.g., foraging and anti-predator behavior) may be important in the health of an animal. Further research is needed to understand the proximate relationship between testosterone and longevity, as well as the physiological and behavioral factors that lead captive mouse lemurs to early senescence compared to their wild counterparts, and to determine if aging in *Microcebus rufus* and other *Microcebus* species is comparable.

## Supporting Information

File S1 **This file includes additional details on the dental impressions and tooth wear analysis, as well as goodness-of-fit results for the mark-recapture survival analysis.** We also include tables of age-specific capture information, dental wear measurements of individuals, estimated regression slopes of tooth wear, predicted ages of captured individuals, dental wear rates compared between the sexes, model selection results of the survival analysis, model averaged estimates of age-specific survival, and fecal testosterone values of the sexes. We also include figures of infrared images of mouse lemurs, dental wear measurements of teeth, and variation in recaptures by age. **Figure S1**, Examples of the infrared images taken to examine for cataracts. **Figure S2**, Method of dental wear measurements. **Figure S3**, Recaptures within each season show no increased frequency of captures by age. **Table S1**, Number of individuals captured annually in each age class. **Table S2**, Dental wear measurements from 2008–2010. **Table S3**, Individual lemurs captured 3 or more consecutive years. **Table S4**, Predicted ages based on dental wear compared to minimum possible ages based on trapping data. **Table S5**, Dental wear rates do not differ between the sexes. **Table S6**, Model selection statistics for closed robust capture-recapture analyses of *Microcebus rufus*. **Table S7**, Model-averaged annual survival probability estimates and standard errors. **Table S8**, Male and female fecal Testosterone values (ng/g).
(PDF)
