# Supplementary material for: Teeth, Sex, and Testosterone: Aging in the World's Smallest Primate
Source: PLoS One. 2014 Oct 29;9(10):e109528. doi: 10.1371/journal.pone.0109528 (PMC4212904; doi:10.1371/journal.pone.0109528)
Supplement: File S1 — This file includes additional details on the dental impressions and tooth wear analysis, as well as goodness-of-fit results for the mark-recapture survival analysis. We also include tables of age-specific capture information, dental wear measurements of individuals, estimated regression slopes of tooth wear, predicted ages of captured individuals, dental wear rates compared between the sexes, model selection results of the survival analysis, model averaged estimates of age-specific survival, and fecal testosterone values of the sexes. We also include figures of infrared images of mouse lemurs, dental wear measurements of teeth, and variation in recaptures by age. Figure S1, Examples of the infrared images taken to examine for cataracts. Figure S2, Method of dental wear measurements. Figure S3, Recaptures within each season show no increased frequency of captures by age. Table S1, Number of individuals captured annually in each age class. Table S2, Dental wear measurements from 2008–2010. Table S3, Individual lemurs captured 3 or more consecutive years. Table S4, Predicted ages based on dental wear compared to minimum possible ages based on trapping data. Table S5, Dental wear rates do not differ between the sexes. Table S6, Model selection statistics for closed robust capture-recapture analyses of Microcebus rufus. Table S7, Model-averaged annual survival probability estimates and standard errors. Table S8, Male and female fecal Testosterone values (ng/g). (PDF) [file pone.0109528.s001.pdf]

Supporting Information

Dental impressions and tooth wear analysis. Linear measurements of exposed dentine on the metaconid and the hypoconid of the second mandibular molar were taken from the 15x images using Image Pro Plus Software. In addition, prepared dental molds were scanned using Nextec Hawk 3D laser scanner at 20um resolution to better visualize wear, then the 3-D scans were analyzed for orientation patch counts (OPC) using Amira software.

For the hypoconid wear, a line was drawn connecting the metaconid and the entoconid (along the entocristid) and dentine exposure of the hypoconid was measured by drawing a perpendicular line from the entocristid-line through the hypoconid (showing maximum wear, highlighted in yellow). For the metaconid wear, a line was drawn connecting the hypoconid and the entoconid (along the postcristid) and dentine exposure of the metaconid was measured by drawing a perpendicular line from the postcristid-line through the metaconid (showing maximum wear, highlighted in orange). Whereas the entoconid showed measurable wear, the hypoconid and metaconid provided the most consistent wear measurements (The R^2 values for hypoconid, metaconid, and entoconid were 0.932, 0.887, and 0.779 respectively). The wear on the protoconid became difficult to measure in worn teeth. While other cusps also showed wear, we found the metaconid and the hypoconid to be most consistently worn in these small teeth (Supp. Fig. 1). The initial tabulations of results show that wear is largely linear from capture to capture irrespective of the stage of wear (Fig. 1b, Supp. Table 1).

Survival Analysis. There is no formal goodness-of-fit statistic for the robust design with covariates, so we collapsed secondary capture histories within each primary period to form an open capture-recapture dataset. From this we conducted goodness of fit to evaluate violations of model assumptions of our most general model (survival and detection varying by age*sex*year) using program RELEASE [6]. The overall test (TEST1) of homogeneity of survival and capture probabilities by group indicated no lack of fit (Overall P=0.9961). We also used TEST3 of program RELEASE to specifically test for transients that may bias the assumption of homogeneity of survival by group; we found no support for transients (Overall P=0.7707). These results suggested that our general model was appropriate for the data.

The robust design includes temporary emigration parameters as a means to consider that some animals in the super-population (animals in the sampling area vicinity that were exposed to trapping efforts) were absent from the study site during sampling during some primary periods. However, we did not observe any individuals that were detected in one year, missed in a following year, and then detected in a subsequent year. Therefore we detected no temporary emigration and fixed these parameters.

Table S1. Number of individuals captured annually in each age class. Individuals classified as age “0” are predicted based on the number of females captured the previous year, assuming that every female gave birth to two offspring, which is the average litter size in brown mouse lemurs.

Age	2008	2009	2010
**0**	39	26	28
**1**	20	21	18
**2**	13	23	17
**3**	9	10	12
**4**	2	4	8
**5**	7	3	3
**6**	1	4	1
**7**	2	1	1
**8**	0	2	0

Table S2. Dental wear measurements from 2008-2010.

Year	Name	Sex	Hypoconid (µm)	Metaconid (µm)	Tooth length (µm)
2008	Irwin	0	74	66	1985
2008	Jumbo-score	0	70	70	1882
2008	Kevin	0	85	66	1956
2008	Ian	0	81	74	1904
2008	Griffindor	0	103	66	1823
2008	Ryan	0	100	74	1845
2008	Rado	0	74	107	1889
2008	Scott	0	92	111	1974
2008	Gob	0	96	107	1967
2008	Turandot	0	100	92	1845
2008	Samson	0	107	92	1867
2008	Rigoletto	0	103	100	1771
2008	Rudolpho	0	103	111	1867
2008	Sam	0	103	122	1845
2008	Mark	0	122	118	1897
2008	Michael	0	114	148	1963
2008	Harley	0	129	125	1838
2008	Mamy	0	122	140	1863
2008	Aristide	0	92	162	1793
2008	Napolean	0	151	107	1812
2008	Pascal	0	148	137	1911
2008	Don	0	148	148	1985
2008	Ralala	0	125	148	1815
2008	Kerry	0	140	129	1779

2008	Lanto	0	137	188	1786
2008	Kristopher	0	170	185	1926
2008	Luka	0	192	218	1889
2008	Barry-licious	0	192	214	1860
2008	Erik	0	196	207	1804
2008	Randy	0	214	210	1867
2008	Toky	0	255	214	2022
2008	Randy	0	214	218	1860
2008	Ismael	0	203	225	1753
2008	Maeybe	1	52	66	1970
2008	Reychell	1	77	66	2306
2008	Lena	1	74	66	1963
2008	Hyacinth	1	77	63	1934
2008	Erin	1	81	59	1875
2008	Hufflepuff	1	74	96	2037
2008	Leila	1	85	74	1875
2008	Violetta	1	70	81	1697
2008	Mary	1	100	81	1756
2008	Brunhilda	1	129	122	1974
2008	Elphaba	1	151	114	2007
2008	Carmen	1	122	114	1720
2008	Manoli	1	155	111	1919
2008	Preciosa	1	125	155	1897
2008	Govinda	1	129	148	1734
2008	Diggy	1	114	151	1653
2008	Claudia	1	111	181	1797
2008	Peggy	1	103	236	1948
2008	Jacqueline	1	162	251	1875
2008	Stacey	1	236	306	1904
2009	Banghra	1	59	100	1934
2009	Raozy	1	92	70	1934
2009	Sasha	1	59	107	1860
2009	Miora	1	48	129	1934
2009	Obamanikwa	1	85	92	1926
2009	Sweet Potato	1	66	111	1867
2009	Iiris	1	111	92	1882
2009	Lalao	1	89	129	1993
2009	Ravo	1	81	137	1849
2009	Vienna	1	59	173	1875
2009	Jessikwa	1	107	129	1882
2009	Leila	1	103	140	1919
2009	Mary	1	118	114	1830
2009	Ravenclaw	1	89	196	2033
2009	Mija	1	96	155	1771
2009	Preciosa	1	137	170	1937

2009	Brunhilda	1	140	162	1849
2009	Carmen	1	133	148	1672
2009	Persephone	1	125	159	1897
2009	Violetta	1	114	155	1871
2009	Govinda	1	177	155	1897
2009	Claudia	1	155	218	1779
2009	Elphaba	1	177	251	1970
2009	Jacqueline	1	192	284	1926
2009	Fern Gully	1	207	446	1970
2009	Queenie	1	310	347	2011
2009	Adafi	0	59	70	2162
2009	Obama	0	85	63	1889
2009	Akondro	0	81	66	1815
2009	Harry Potter	0	85	89	2066
2009	Sharky	0	92	59	1764
2009	Gandalf	0	77	114	2096
2009	Ananas	0	81	100	1948
2009	Zoolander	0	85	100	1963
2009	Rajao	0	74	96	1779
2009	Pappu	0	77	96	1793
2009	Banana	0	92	77	1734
2009	Shah Rukh	0	92	107	1934
2009	Punjab	0	85	107	1793
2009	Solofo	0	74	125	1749
2009	Theo	0	114	107	1937
2009	Kumar	0	92	118	1827
2009	Zac Efron	0	96	114	1827
2009	Zohdy	0	125	111	2007
2009	Harold	0	103	122	1871
2009	Johary	0	137	107	2022
2009	Hurley	0	92	133	1797
2009	Blarney	0	103	122	1793
2009	Jersey	0	114	125	1897
2009	Mugatu	0	140	114	1996
2009	Jernvall	0	107	129	1804
2009	Scott	0	114	148	1915
2009	Ian	0	92	107	1812
2009	Ryan	0	103	122	1867
2009	Borat	0	92	162	1797
2009	Nordiny	0	107	155	1793
2009	Godzilla	0	133	221	2133
2009	Kerry	0	148	148	1771
2009	Napolean	0	159	125	1852
2009	Ralala	0	170	148	1852
2009	Mamy	0	170	188	1908

2009	Mark	0	199	173	1815
2009	Pascal	0	162	210	1815
2009	Aristide	0	125	314	1878
2009	Michael	0	177	240	1952
2009	Boris	0	273	232	1930
2009	Randy	0	225	251	1875
2009	Toky	0	354	188	2048
2009	Ismael	0	225	332	1849
2010	Addie	1	80	60	1830
2010	Alessandra	1	80	70	1810
2010	Barble	1	120	110	1850
2010	Brunhilda	1	130	216	1880
2010	Charlotte	1	80	90	1930
2010	Claudia	1	190	220	1810
2010	Digit	1	70	120	1870
2010	Gaga	1	70	80	1840
2010	Iiris	1	49	82	1900
2010	Juliet	1	70	83	1907
2010	Kate	1	58	88	1861
2010	Lalao	1	100	140	1900
2010	Libby	1	90	91	1885
2010	Liza	1	74	91	1836
2010	Lotta	1	69	95	1850
2010	Lolita	1	93	115	1784
2010	Lourdes	1	100	155	1706
2010	Marwa	1	125	140	1827
2010	Miora	1	90	145	1874
2010	Onja	1	100	140	1899
2010	Persephone	1	185	220	1958
2010	Ravenclaw	1	125	140	1950
2010	Turandot	1	135	210	1930
2010	Adafi	0	90	110	2070
2010	Adonis	0	90	120	1700
2010	Ben	0	110	130	1740
2010	Blarney	0	110	170	1800
2010	Borat	0	170	150	1810
2010	Christopher	0	110	110	1910
2010	Esquelito	0	92	102	1820
2010	Fanks	0	90	90	1880
2010	Garth	0	170	220	1950
2010	Gandalf	0	118	119	1762
2010	Gonzales	0	99	90	1740
2010	Ian	0	120	160	1860
2010	Igor	0	68	143	1906
2010	Jersey	0	130	138	1858

2010	Jernvall	0	133	140	1805
2010	Jin	0	88	116	1873
2010	Jeff	0	58	123	1790
2010	Kahn	0	97	87	1956
2010	Kerry	0	170	200	1784
2010	Laurent	0	134	111	1868
2010	Mamy	0	178	295	1977
2010	Manatena	0	90	130	1734
2010	Mangovetra	0	110	137	1858
2010	Mark	0	188	220	1802
2010	Napolean	0	183	178	1799
2010	Pappu	0	62	140	1765
2010	Rachootin	0	82	83	1931
2010	Rajao	0	89	120	1828
2010	Scott	0	132	204	1862
2010	Sawyer	0	145	178	1972
2010	Sherman	0	248	208	1870
2010	Solofo	0	142	166	1851
2010	Taz	0	183	170	1855
2010	Teemu	0	112	137	1880
2010	Zohdy	0	160	173	2058
2010	Zoolander	0	112	170	2025
2010	Ole	0	168	139	2025

Table S3. Individual lemurs captured 3 or more consecutive years. In the table below, the linear regression slopes and reduced major axis (RMA) slopes are presented for individuals captured 3 or more consecutive years. The R^2 values show intra-individual linear wear. Raw dental measurements are in table S9.

**Name**	**Linear slope**	R^2	**RMA slope**
**Ingrid**	616.48	0.90	650.99
**Anja**	654.84	0.96	668.33
**Stacey**	650.73	0.97	661.04
**Marcela**	706.05	0.99	707.16
**Carla**	490.51	0.95	504.27
**Claudia**	731.20	0.98	740.42
**Govinda**	789.99	0.95	809.83
**Jaqueline**	676.13	0.95	691.89
**Preciosa**	1177.43	0.98	1190.16
**Jenna**	767.91	0.99	772.70
**Sherry**	676.82	0.55	916.12
**Aristide**	355.36	0.80	397.49
**Ismael**	611.50	0.94	629.70
**Kerry**	966.35	0.98	976.78
**Mamy**	582.78	0.96	594.87
**Mark**	447.03	0.95	459.36
**Napolean**	900.55	0.93	932.56
**Pascal**	569.15	0.96	581.04
**Ralala**	782.25	0.98	790.72
**Scott**	799.68	0.99	803.66
**Mickey**	684.35	0.97	695.84
**Ziggy**	1010.69	0.99	1017.34
**Loco**	549.62	0.97	558.10

Table S4. Predicted ages based on dental wear compared to minimum possible ages based on trapping data. The 17 individuals that had underestimated ages are highlighted in yellow.

Predicted	Minimum	Frequency		Predicted	Minimum	Frequency
1	1	62		9	5	0
2	1	43		1	6	0
3	1	9		2	6	0
4	1	5		3	6	0
5	1	5		4	6	0
6	1	2		5	6	0
7	1	0		6	6	0
8	1	1		7	6	0
9	1	0		8	6	1
1	2	9		9	6	0
2	2	21		1	7	0
3	2	15		2	7	0
4	2	4		3	7	0
5	2	3		4	7	0
6	2	2		5	7	0
7	2	0		6	7	0
8	2	0		7	7	0
9	2	0		8	7	0
1	3	0		9	7	0
2	3	2		1	8	0
3	3	12		2	8	0
4	3	5		3	8	0
5	3	4		4	8	0
6	3	2		5	8	0
7	3	0		6	8	0
8	3	1		7	8	0
9	3	0		8	8	0
1	4	0		9	8	0
2	4	0		1	9	0
3	4	4		2	9	0
4	4	8		3	9	0
5	4	1		4	9	0
6	4	5		5	9	0
7	4	0		6	9	0
8	4	0		7	9	0
9	4	0		8	9	0
1	5	0		9	9	0
2	5	0				
3	5	0				
4	5	2				
5	5	2				

6	5	0				
7	5	3				
8	5	0				

Table S5. Dental wear rates do not differ between the sexes. The RMA slopes of dental wear in individuals that were captured in 3 or more consecutive years do not differ between the sexes (t=0.66, *P*=0.516)

**Females**	**Males**
650.9992	397.4866
668.3304	629.7099
661.0471	976.7846
707.1551	594.8693
504.2667	459.3571
740.4206	932.56
809.826	581.0368
691.8931	790.7225
1190.16	803.6585
772.6956	695.8414
916.1203	1017.337
	558.098

Table S6. Model selection statistics for closed robust capture-recapture analyses of *Microcebus rufus*.

Model[1]	AIC_c	ΔAIC_c	AIC_c Weight	Model Likelihood	No. Parameters	Deviance
$S(age(T_{L)} + sex)\ \pi(.)\ p(het + sex)$	2315.134	0.000	0.395	1.000	7	2300.856
$S(age(T_L))\ \pi\ (.)\ p(het + sex)$	2315.534	0.400	0.323	0.819	6	2303.326
$S(age(T_{Q)} + sex)\ pi(.)\ (het + sex)$	2317.137	2.003	0.145	0.367	8	2300.779
$S(age(T_Q))\ \pi\ (.)\ p(het + sex)$	2317.593	2.459	0.116	0.292	7	2303.315
$S(age + sex)\ \pi\ (.)\ p(het + sex)$	2321.863	6.730	0.013	0.035	11	2299.202
$S(age)\ \pi\ (.)\ p(het + sex)$	2323.256	8.122	0.007	0.017	10	2302.706
$S(age + sex)\ (.)\pi\ (.)\ p(het)$	2329.424	14.290	0.000	0.000	10	2308.874
$S(.)\pi\ (.)\ p(het + sex)$	2339.377	24.244	0.000	0.000	5	2329.229
$S(sex)\ (.)\pi\ (.)\ p(het + sex)$	234.312	25.178	0.000	0.000	6	2328.104
$S(.)\pi\ (year)\ p((het*year) + sex)$	234.891	25.758	0.000	0.000	13	2313.974
$S(year)\ \pi\ (.)\ p(het + sex)$	2342.593	27.460	0.000	0.000	7	2328.315
$S(year + sex)\ \ \pi\ (.)\ p(het + sex)$	2343.68	28.546	0.000	0.000	8	2327.322
$S(.)\ \ \pi\ (year)\ p(year * het)$	2345.632	30.499	0.000	0.000	12	2320.849
$S(.)\ \ \pi\ (.)\ p(het)$	2345.8.2	30.668	0.000	0.000	4	2337.703
$S(sex)\ \ \pi\ (.)\ p(het)$	2347.61	32.477	0.000	0.000	5	2337.462
$S(year)\ \ \pi\ (.)\ p(het)$	2348.919	33.785	0.000	0.000	6	2337.462
$S(.)\ \ p(age * year)$	2428.446	113.313	0.000	0.000	21	2336.711
$S(.)\ \ p(age + sex)$	2435.551	120.418	0.000	0.000	7	2384.071
$S(.)\ \ p(age)$	2441.522	126.389	0.000	0.000	6	2421.274
$S(.)\ \ p(year + sex)$	2445.635	130.501	0.000	0.000	6	2429.314
$S(.)\ \ p((age * year) + sex)$	2446.964	131.831	0.000	0.000	22	2433.427
$S(.)\ \ p(year)$	2459.657	144.523	0.000	0.000	5	2400.356
$S(.)\ \ p(sex)$	2466.131	150.997	0.000	0.000	3	2449.509
$S(.)\ \ p(.)$	2478.495	163.362	0.000	0.000	2	2460.072

[1]Survival, S, is modeled as varying by *year*, *sex*, *age*, *age* and *sex (e.g age + sex)*, age varying as a linear trend ($age(T_L)$), age varying as a quadratic trend ($age(T_Q)$), a trend and *sex*, or otherwise as constant (.). The probability of temporary emigration (γ) was consistent across all models as $\gamma'=1$ and $\gamma''=0$ to indicate no movement, due to data and lack of support for other variants in initial investigations. Probability of initial capture (*p*) and subsequent (*c*) recapture are equivalent for all models because the start of trapping occurred before the sampling period used for these analyses, thus a behavioral effect was unlikely. *p* and thus *c* are modeled as constant (.), varying by *sex*, *year*, heterogeneity (*het*), *age*, or mixed combinations. Heterogeneity was modeled using a finite mixture model of two-groups, where the proportion in each group was either constant or varied by *year*.

Table S7. Model-averaged annual survival probability estimates and standard errors. The numbers in bold are the survival probabilities calculated from mark recapture data, the numbers not bolded are the predicted survival probabilities based on the model trend.

Age	Annual Survival Probability	SE
**1-2**	**0.804**	**0.090**
**3**	**0.739**	**0.073**
**4**	**0.667**	**0.075**
**5**	**0.591**	**0.083**
**6**	**0.509**	**0.104**
7	0.388	
8	0.319	
9	0.251	
10	0.182	
11	0.113	
12	0.045	

Table S8. Male and female fecal Testosterone values (ng/g). We found no significant difference in testosterone between the sexes. When controlling for date, using generalized linear mixed effect models (GLMM) with a gamma distribution and a log link, we found no significant difference between male and female fecal T levels $F(1,81)=0.02$, $P=0.90$. Numbers in parentheses are the numerator and denominator degrees of freedom for corresponding F-values.

**Males**	**Females**
n 339	*n* 201
Mean:12.11	Mean: 12.87
s.d: 11.14	s.d: 12.28

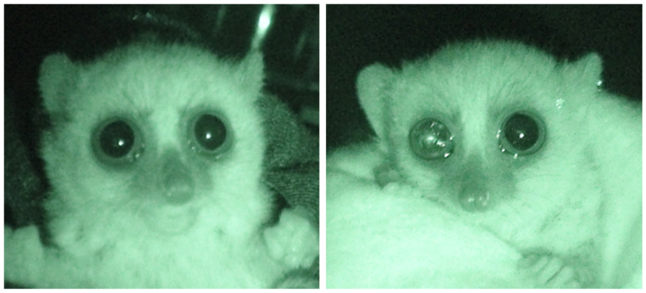

Figure S1. Examples of the infrared images taken to examine for cataracts. An individual without any ocular pathologies (left) and the only individual captured with any ocular pathology. This infection can be seen as a cloudy region on the lens of the animal's right eye (right).

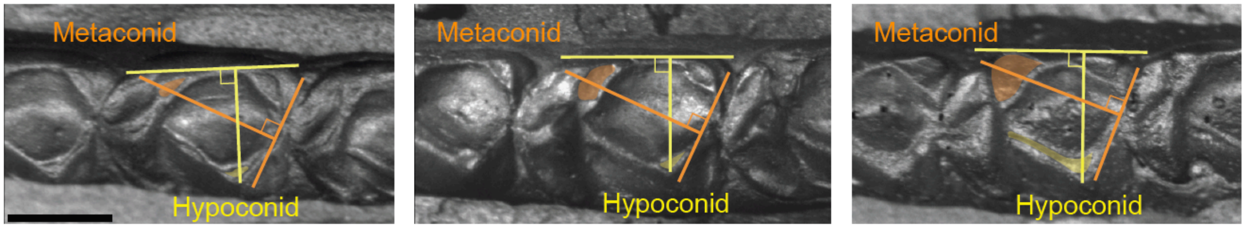

Figure S2. Method of dental wear measurements. Scale bar, 1 mm.

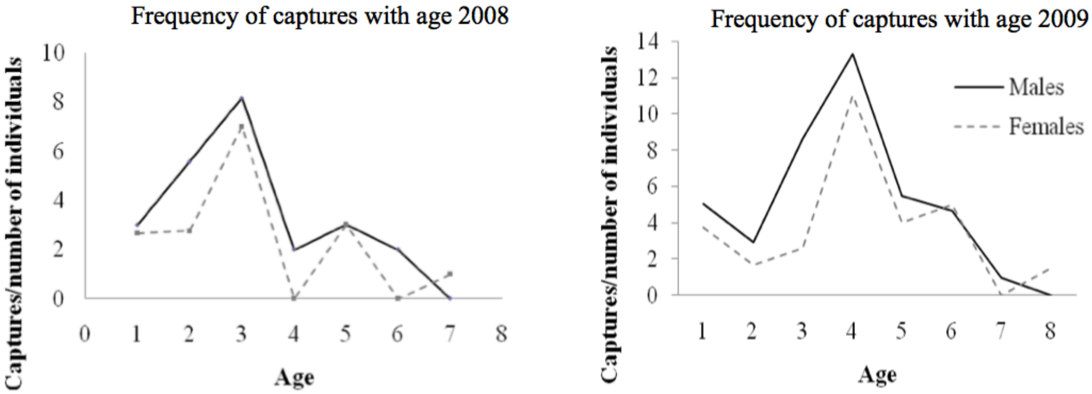

Figure S3. Recaptures within each season show no increased frequency of captures by age. We plotted the number of captures over the number of individuals in each age class (y-axis) against the age classes (x-axis).
